# Disproportionality analysis of drug-related interstitial lung disease in patients with head and neck squamous cell carcinoma: a signal mining study

**DOI:** 10.3389/fimmu.2026.1858004

**Published:** 2026-07-13

**Authors:** Jie Ju, Zhongfen Liu, Lin Cao, Lina Xia, Pei Zhang, Yanfei Wang

**Affiliations:** 1 Key Laboratory of Carcinogenesis and Translational Research (Ministry of Education), Day Oncology Unit, Peking University Cancer Hospital and Institute, Beijing, China; 2 Key Laboratory of Carcinogenesis and Translational Research (Ministry of Education), Department of Supportive Care, Peking University Cancer Hospital and Institute, Beijing, China

**Keywords:** cetuximab, disproportionality analysis, head and neck squamous cell carcinoma, interstitial lung disease, PD-1/PD-L1 inhibitor

## Abstract

**Background:**

With the widespread use of novel therapeutic agents, an increasing number of drugs have been confirmed to be associated with interstitial lung disease (ILD). However, real-world evidence regarding ILD reporting signals in patients with head and neck squamous cell carcinoma (HNSCC) remains limited.

**Methods:**

We performed a signal mining study using data from the FDA Adverse Event Reporting System (FAERS) database from Q1 2004 to Q1 2025. Eligible adverse event reports related to ILD in HNSCC patients were included. Disproportionality analysis was performed using the combination of reporting odds ratio (ROR) and information component (IC). Time to onset (TTO) was analyzed using descriptive statistics and Weibull distribution modeling.

**Results:**

A total of 513 patients with ILD related to HNSCC treatment in the FAERS database were included. The median age was 68.00 years (IQR 60.00-75.00), the median weight was 56.00kg (IQR 47.80-68.00), and a total of 42 drugs were involved. Based on ROR and IC, three drugs showed definite positive ILD signals: cetuximab [ROR = 1.46 (95%CI: 1.22-1.74), IC = 0.36 (95%CI: 0.12-0.60)], nivolumab [ROR = 1.67 (95%CI: 1.35-2.06), IC = 0.61 (95%CI: 0.30-0.90)], and pembrolizumab [ROR = 2.39 (95%CI: 1.93-2.96), IC = 1.06 (95%CI: 0.74-1.35)]. In addition, etoposide showed a positive ROR [ROR = 4.51 (95%CI: 1.41-14.43)] but a negative IC [IC = 2.11 (95%CI: -0.26-2.72)]. Drug class analysis revealed that anti-EGFR monoclonal antibodies [ROR = 1.45 (95%CI: 1.21-1.72), IC = 0.35 (95%CI: 0.11-0.59)] and PD-1/PD-L1 inhibitors [ROR = 2.14 (95%CI: 1.80-2.54), IC = 0.72 (95%CI: 0.49-0.95)] were classes with positive safety signals for ILD. The median TTO was 32.00 days (IQR 14.00-73.50), α=61.70 (95%CI 52.98-71.85), β=0.83 (95%CI 0.76-0.90), and ILD was mainly characterized by early onset.

**Conclusion:**

ILD reports associated with HNSCC treatment were mainly concentrated among anti-EGFR monoclonal antibodies and PD-1/PD-L1 inhibitors, with the early treatment stage representing a critical window that warrants strengthened monitoring for ILD in clinical practice. Given spontaneous reporting data, causality cannot be confirmed and further verification is required.

## 1 Introduction

Head and neck squamous cell carcinoma (HNSCC) is one of the most common malignant tumors worldwide, with approximately 891000 new cases globally and 146000 new cases in China in 2022, and the incidence rate is significantly higher in males than in females (1, 2). With the understanding of the molecular mechanisms of tumors, the systemic treatment model for HNSCC has gradually shifted from traditional chemotherapy to precision treatment strategies centered on targeted therapy and immunotherapy. These novel therapies have also brought new challenges to drug safety.

Drug-induced interstitial lung disease (ILD) is one of the severe adverse reactions that require high vigilance. Characterized by interstitial inflammation and fibrotic changes in the lung, it has a complex etiology and poor prognosis, with a mortality rate as high as 20% (3). HNSCC patients usually have a long smoking history, some are complicated with underlying lung diseases such as chronic obstructive pulmonary disease, and many receive radiotherapy, making them more susceptible to drug-related ILD during treatment. Such adverse events may not only lead to the interruption of treatment but also rapidly progress to respiratory failure and even life-threatening conditions. Although the overall incidence of ILD is low, the proportion of severe cases is high. Typical clinical manifestations include progressive dyspnea and dry cough, sometimes accompanied by systemic symptoms such as low-grade fever and fatigue.

Previous studies have revealed marked variations in ILD risks among diverse cancer types and therapeutic agents. Current researches are mainly focused on non-small cell lung cancer and melanoma, while studies on HNSCC remain limited, mostly in the form of case reports or small-sample retrospective studies. For example, a study including 201 HNSCC patients treated with cetuximab showed that only 9 cases (4.5%) developed ILD, of which 8 were grade ≥3 severe events (4). With the FDA approval of nivolumab and pembrolizumab in 2016, HNSCC patients have entered the era of immunotherapy. Compared with conventional chemotherapy, immunotherapy has increased the incidence of ILD in HNSCC patients, which is consistent with the results of a pan-cancer study by Baxi et al. (5, 6). A retrospective analysis of 179 HNSCC patients treated with cetuximab and/or PD-1 inhibitors found that the incidence of ILD in patients receiving concurrent PD-1 inhibitor and cetuximab treatment (18.6%) was higher than that in patients receiving cetuximab alone (7.9%) and those receiving PD-1 inhibitor alone (11.4%), although this increase in risk was not statistically significant (7).

Since drug-related ILD mostly emerges after marketing, randomized controlled trials (RCTs), limited by strict inclusion and exclusion criteria as well as limited observation duration, cannot fully reflect the occurrence of adverse events in the real world. In contrast, real-world studies have the advantages of large sample size, high population heterogeneity, and long follow-up time, which are more conducive to identifying rare or severe adverse reactions and more truly reflecting the occurrence frequency and characteristics of adverse events in clinical practice. However, real-world studies on drug-related ILD in HNSCC are still scarce, and a systematic risk assessment is lacking. In addition, disproportionality analysis methods have been widely used for drug safety signal detection. Among them, the reporting odds ratio (ROR) and information component (IC) have become the most commonly used disproportionality analysis methods in spontaneous reporting databases due to their core advantages of simple calculation and clear statistical significance. In summary, based on large-scale real-world data from the FAERS database, this study adopted the combined ROR and IC method to systematically evaluate the ILD reporting signals of drugs for HNSCC treatment, screen agents with positive reporting signals, and provide evidence for clinical management and drug regulation.

## 2 Methods

### 2.1 Data source

The disproportionality analysis of this study was based on the FAERS database, a comprehensive pharmacovigilance database managed by the FDA that collects adverse event reports from healthcare professionals, consumers, and pharmaceutical companies. The database contains patient demographic and administrative information (DEMO), drug information (DRUG), adverse event information (REAC), patient outcomes (OUTC), report sources (RPSR), drug treatment start and end times (THER), and medication indications (INDI), among other data. All adverse events were coded using the preferred term (PT) according to version 28.0 of the Medical Dictionary for Regulatory Activities (MedDRA). ILD-related adverse events were identified using the narrow Standardized MedDRA Query (SMQ) for ILD. This standardized composite query includes 46 relevant PTs, such as pulmonary fibrosis, immune-mediated lung disease and Idiopathic interstitial pneumonia.

Data on drug adverse events related to HNSCC treatment from the first quarter of 2004 to the first quarter of 2025 were retrieved from the FAERS database. All FAERS reports of the 42 drugs in patients with HNSCC, including 39 antineoplastic drugs (12 chemotherapy drugs, 19 targeted drugs and 8 immune checkpoint inhibitors) and 3 supportive drugs in cancer treatment. Only primary suspect drugs were included in the disproportionality analysis.

Given the spontaneous reporting model of the FAERS database, which has the problems of duplicate reports, withdrawn or deleted reports, this study strictly performed data cleaning in accordance with the guidance documents on the FDA official website. The CASEID, FDA_DT, and PRIMARYID fields of the DEMO table were selected and sorted by CASEID, FDA_DT, and PRIMARYID. For reports with the same CASEID, the one with the largest FDA_DT value was retained; for reports with the same CASEID and FDA_DT, the one with the largest PRIMARYID value was retained. In addition, a list of deleted reports has been included in each quarterly data package since the first quarter of 2019, and reports were excluded according to the CASEID in the deleted report list after data deduplication.

A total of 22775812 patient-reported data were obtained from the FAERS database in this study. According to the FDA deduplication rules, 3837944 cases of duplicate reporting data from the same patient were excluded, leaving 18937868 patients with 56321150 adverse event reports after deduplication. Further data screening was performed, and a total of 49053 reports from 16689 patients were included for analysis. Screening was conducted using PT in the MedDRA dictionary, and 513 patients with ILD were identified, involving 540 ILD reports.

### 2.2 Statistical analysis

For disproportionality analysis using ROR and IC, the criteria for signal generation were defined as “ILD reports ≥3, the lower limit of the 95% CI of ROR >1” and “the lower limit of the 95% CI of IC >0”. The ROR or IC value reflects the magnitude of the statistical association between the drug and ILD. The higher ROR or IC value indicates a stronger disproportionality signal, rather than a higher clinical risk.

Descriptive analysis was conducted on the clinical characteristics of the cases. The time from the start of medication to the occurrence of ILD was defined as time to onset (TTO), and its distribution was described using the median and interquartile range (IQR). The scale parameter (α) and shape parameter (β) were calculated using the Weibull distribution model, where the scale parameter α reflects the timing of event occurrence (a larger α indicates a later event onset, and a smaller α indicates an earlier event onset), and the shape parameter β reflects the change rule of risk over time (β>1 indicates an increasing risk; β=1 indicates a constant risk; β<1 indicates a decreasing risk). The Kaplan-Meier method was used to display the distribution of onset times among cases with available data. The Wilcoxon test was used for intergroup comparison, and the Kruskal-Wallis test was used for multiple group comparison. The P-value <0.05 was considered statistically significant. In addition, disproportionality analysis was performed only on cases reported by healthcare professionals to further verify the reliability of the results. All statistical analyses were completed using SAS version 9.4 software.

## 3 Results

### 3.1 Descriptive analysis

A total of 513 HNSCC patients with ILD were included, among whom 373 were male (72.71%) and 84 were female (16.37%). The median age of the patients was 68.00 years (IQR: 60.00-75.00), and the median body weight was 56.00 kg (IQR: 47.80-68.00). The top three countries of patient origin were Japan (190 cases, 37.04%), Germany (122 cases, 23.78%), and the United States (88 cases, 17.15%). Among them, 510 cases had severe outcomes (99.42%), 307 cases were hospitalized (59.84%), and 214 cases died (41.72%). The specific situation is shown in **Table 1**. Among the three drugs with definite ILD signals, the proportions of fatal reported cases for ILD associated with nivolumab, pembrolizumab, and cetuximab were 43.24% (48/111), 38.32% (41/107), and 40.20% (80/199), respectively.

**Table 1:** Patient characteristics of patients with ILD induced by drugs.

Characteristics	Total no. (%)
Gender	
N (Missing)	56 (10.92)
Male	373 (72.71)
Female	84 (16.37)
Age (years)	
N (Missing)	438 (75)
Mean (SD)	66.43 (11.50)
Median (Q1, Q3)	68.00 (60.00, 75.00)
Min, Max	18.00, 90.00
Reporting year	
2004	4 (0.78)
2005	5 (0.97)
2006	6 (1.17)
2007	5 (0.97)
2008	2 (0.39)
2009	9 (1.75)
2010	6 (1.17)
2011	11 (2.14)
2012	5 (0.97)
2013	7 (1.36)
2014	15 (2.92)
2015	28 (5.46)
2016	27 (5.26)
2017	40 (7.80)
2018	60 (11.70)
2019	51 (9.94)
2020	51 (9.94)
2021	55 (10.72)
2022	43 (8.38)
2023	31 (6.04)
2024	38 (7.41)
2025	14 (2.73)
Reporter	
Consumer	143 (27.88)
Not Specified	6 (1.17)
Other health-professional	58 (11.31)
Pharmacist	59 (11.50)
Physician	247 (48.15)
Reported countries (Top 6)	
Japan	190 (37.04)
Germany	122 (23.78)
United States of America	88 (17.15)
France	25 (4.87)
China	15 (2.92)
Italy	13 (2.53)
Serious report	
Serious	510 (99.42)
Non-Serious	3 (0.58)
Outcome	
Life-Threatening	88 (17.15)
Hospitalization - Initial or Prolonged	307 (59.84)
Disability	25 (4.87)
Death	214 (41.72)
Congenital Anomaly	0 (0.00)
Required Intervention to Prevent Permanent Impairment/Damage	2 (0.39)
Other	346 (67.45)
Time to onset (day)	
N (Missing)	280 (233)
Mean (SD)	67.75 (112.13)
Median (Q1, Q3)	32.00 (14.00, 73.50)
Min, Max	0.00, 1101.00

SD, Standard Deviation; Q1, First Quartile; Q3, Third Quartile.

Results were displayed as number (%) if not particularly stated.

### 3.2 Disproportionality analysis

**Figure 1:**
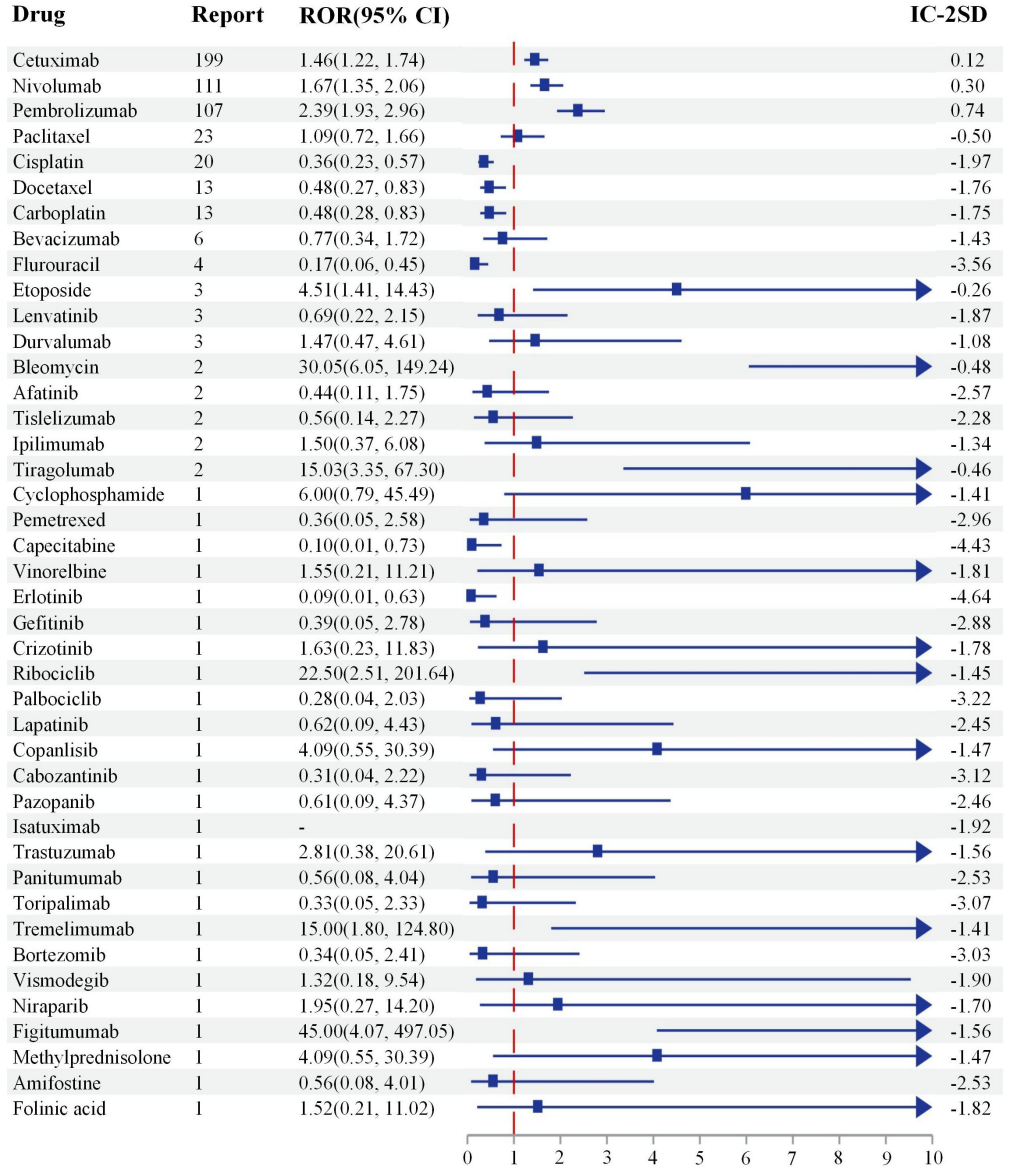
Forest plot showing the disproportionality analysis of drug-related ILD in HNSCC treatment based on ROR and IC. ROR, Reporting Odds Ratio; CI, Confidence Interval; IC, Information Component; IC-2SD, IC minus 2 standard deviations; EGFR, Epidermal Growth Factor Receptor; PD-1, Programmed Death-1; PD-L1, Programmed Death Ligand-1.

ILD reports related to HNSCC treatment in the FAERS database involved 42 drugs, and their ROR and IC values are shown in **Figure 1**, and the corresponding 2×2 contingency tables are provided in the **Supplementary Table 1**. Based on the combined ROR and IC criteria, three drugs with definite ILD reporting signals were identified: cetuximab [ROR = 1.46 (95%CI: 1.22-1.74), IC = 0.36 (95%CI: 0.12-0.60)], nivolumab [ROR = 1.67 (95%CI: 1.35-2.06), IC = 0.61 (95%CI: 0.30-0.90)], and pembrolizumab [ROR = 2.39 (95%CI: 1.93-2.96), IC = 1.06 (95%CI: 0.74-1.35)]. Etoposide showed a positive ROR signal [ROR = 4.51 (95%CI: 1.41-14.43)] but a negative IC signal [IC = 2.11 (95%CI: -0.26-2.72)]. Drug class analysis showed that both anti-EGFR monoclonal antibodies [ROR = 1.45 (95%CI: 1.21-1.72), IC = 0.35 (95%CI: 0.11-0.59)] and PD-1/PD-L1 inhibitors [ROR = 2.14 (95%CI: 1.80-2.54), IC = 0.72 (95%CI: 0.49-0.95)] were classes with strong safety signals for ILD. In addition, in the subgroup of patients aged ≥65 years, only pembrolizumab showed positive ROR and IC signals [ROR = 1.87 (95%CI: 1.42-2.47), IC = 0.72 (95%CI: 0.31-1.10)].

### 3.3 Time to onset analysis of ILD

**Figure 2:**
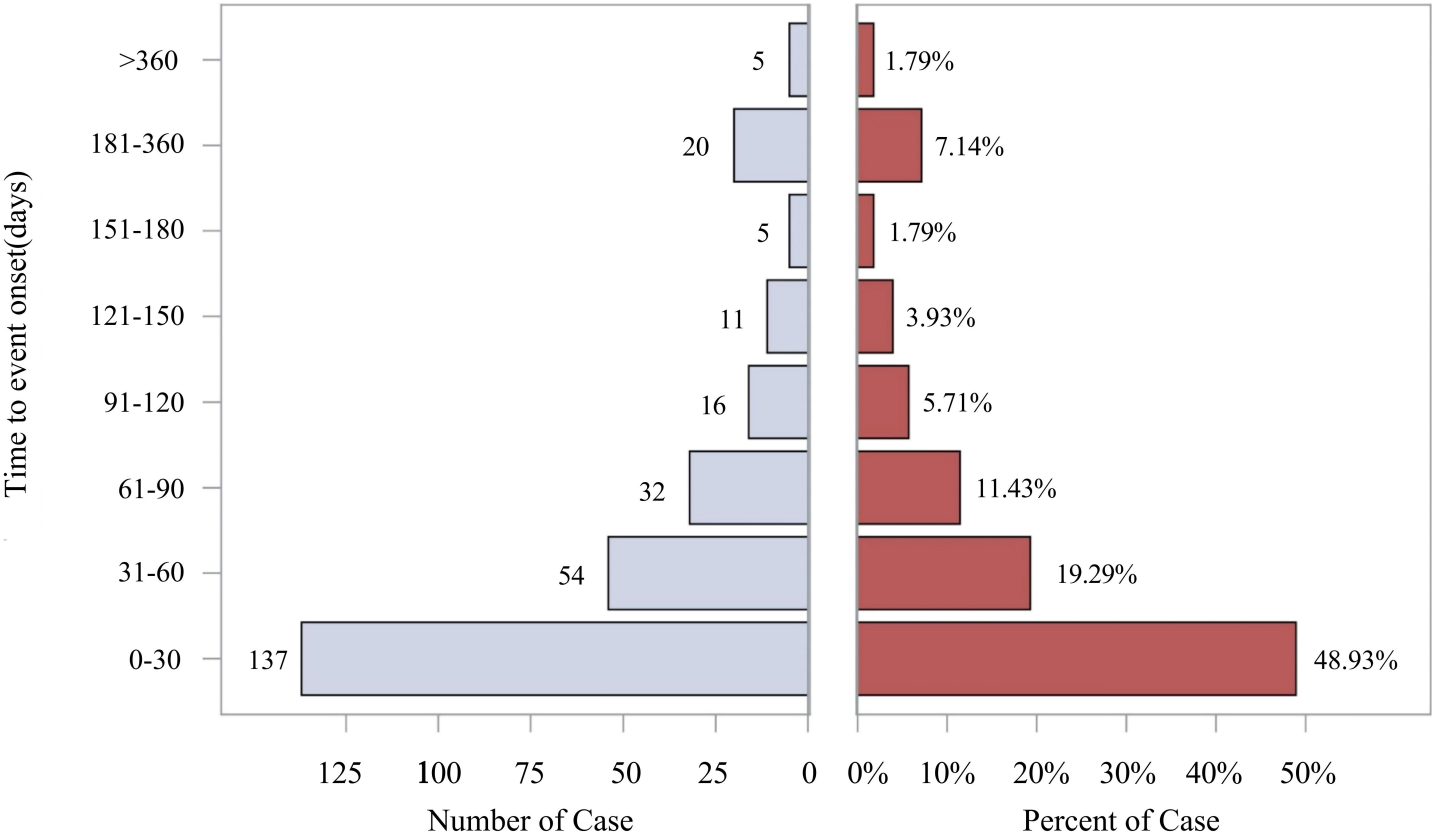
Histogram of TTO of drug-associated ILD in HNSCC patients, with the left panel showing the number of cases and the right panel showing the percentage of cases. TTO, Time to Onset; ILD, Interstitial Lung Disease; HNSCC, Head and Neck Squamous Cell Carcinoma.

A total of 280 valid time to onset (TTO) records were obtained in the study, with a median TTO of 32.00 days (IQR: 14.00-73.50), α=61.70 (95%CI: 52.98-71.85), and β=0.83 (95%CI: 0.76-0.90). Approximately 48.93% (137/280) of ILD cases occurred within 30 days after medication, and 79.64% (223/280) within 90 days after medication, while 5 cases (1.79%) still developed ILD more than 360 days after medication, with the specific distribution shown in **Figure 2**. The above results suggest that drug-related ILD in HNSCC patients is mainly characterized by early onset, but clinical vigilance for late-onset ILD is still highly required.

There were differences in TTO among different drugs (**Table 2**). The median TTO of cetuximab was 29 days (IQR: 14.00-55.00), that of pembrolizumab was 29 days (IQR: 10.00-95.00), and that of nivolumab was 52 days (IQR: 19.00-111.00). The shape parameter β of all three drugs was < 1, also indicating that the early stage of medication is a critical period for ILD risk prevention.

**Table 2:** TTO of ILD for the top 10 drugs ranked by TTO event count.

Drug	TTO event count	TTO (Q1, Q3)	α (95% CI)	β (95% CI)
Cetuximab	103	29.00 (14.00–55.00)	50.99 (39.44–65.91)	0.81 (0.71–0.92)
Nivolumab	69	52.00 (19.00–111.00)	84.39 (65.37–108.95)	0.98 (0.82–1.18)
Pembrolizumab	33	29.00 (10.00–95.00)	62.56 (39.85–98.21)	0.81 (0.62–1.07)
Cisplatin	15	35.00 (14.00–51.00)	51.00 (23.16–112.34)	0.68 (0.47–0.98)
Paclitaxel	10	7.00 (0.00–14.00)	18.27 (9.67–34.50)	1.24 (0.73–2.12)
Docetaxel	8	24.50 (19.50–47.50)	42.73 (24.17–75.52)	1.29 (0.78–2.14)
Carboplatin	4	39.00 (7.50–120.50)	57.24 (16.03–204.36)	0.82 (0.37–1.78)
Bevacizumab	3	24.00 (23.00–65.00)	42.52 (23.84–75.84)	2.08 (0.87–4.95)
Lenvatinib	3	83.00 (15.00–390.00)	151.57 (38.25–600.56)	0.87 (0.35–2.15)
Durvalumab	3	28.00 (25.00–32.00)	29.65 (26.60–33.06)	11.03 (4.51–26.98)

CI, Confidence Interval; Q1, First Quartile; Q3, Third Quartile.

TTO was expressed in days.

**Figure 3:**
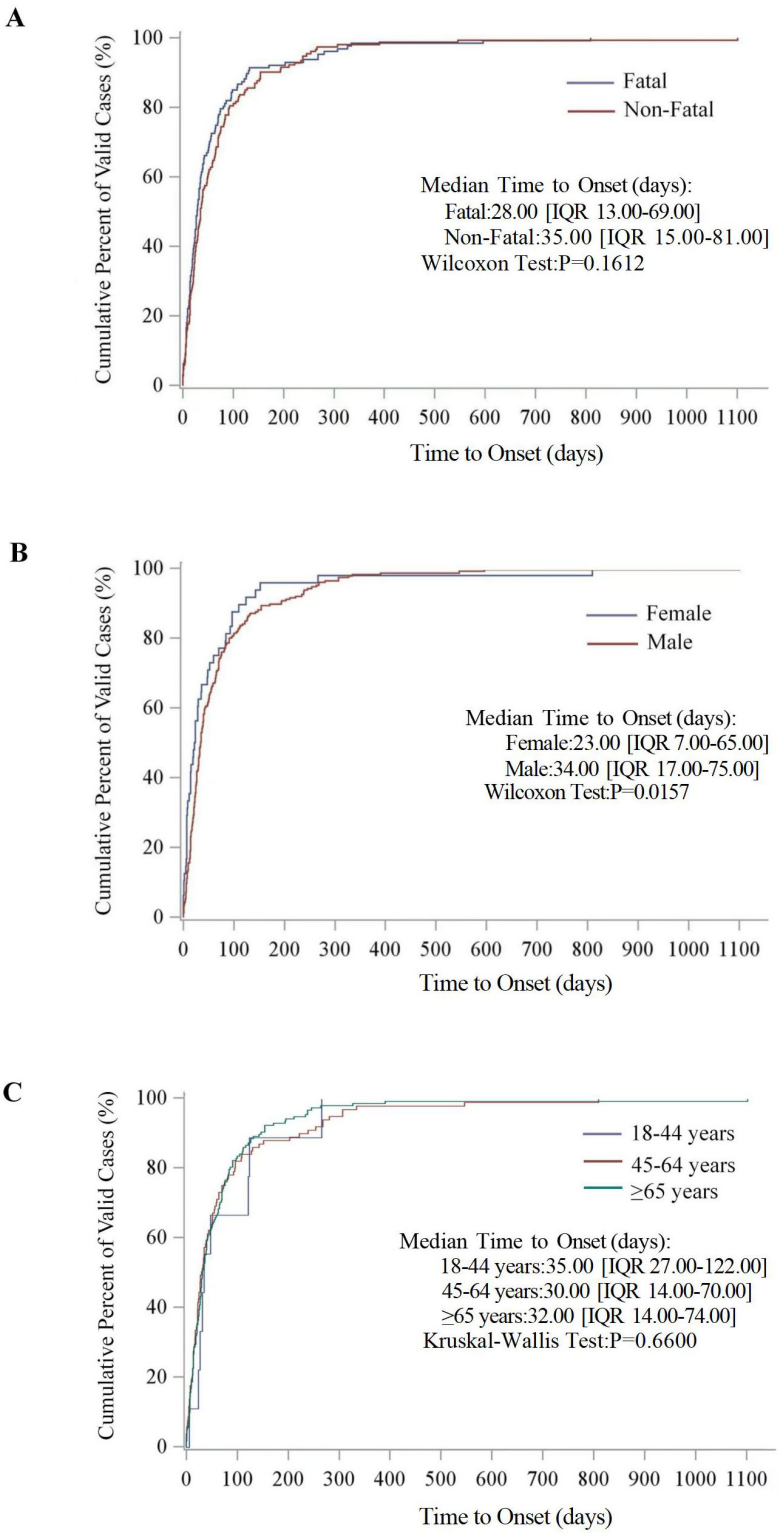
Kaplan-Meier curves for TTO of ILD among cases with available data across different subgroups of HNSCC patients. **(A)** TTO of ILD in fatal and non-fatal cases. **(B)** TTO of ILD in males and females. **(C)** TTO of ILD in different age groups. M, Male; F, Female; TTO, Time to Onset; ILD, Interstitial Lung Disease; HNSCC, Head and Neck Squamous Cell Carcinoma.

Subgroup analysis of different populations showed that the median TTO of non-fatal cases and fatal cases was 35.00 days (IQR: 15.00-81.00) and 28.00 days (IQR: 13.00-69.00), respectively, with no statistically significant difference (Wilcoxon Test: P = 0.1612) (**Figure 3A**). The median TTO of males and females was 34.00 days (IQR: 17.00-75.00) and 23.00 days (IQR: 7.00-65.00), respectively, with a statistically significant difference (Wilcoxon Test: P = 0.0157), which may be related to the epidemiological characteristic of HNSCC with a male predominance (**Figure 3B**). There was no statistically significant difference in TTO among different age groups (Kruskal-Wallis Test: P = 0.6600) (**Figure 3C**).

### 3.4 Disproportionality analysis by healthcare professionals

To improve data quality and reduce erroneous reports, this study excluded cases reported by non-professionals such as consumers, and only performed disproportionality analysis on ILD cases reported by healthcare professionals. The results showed that nivolumab [ROR = 2.10 (95%CI: 1.68-2.63), IC = 0.83 (95%CI: 0.50-1.13)] and pembrolizumab [ROR = 2.84 (95%CI: 2.23-3.62), IC = 1.25 (95%CI: 0.88-1.57)] had definite disproportional association signals in reports from healthcare professionals. Cetuximab [ROR = 1.34 (95%CI: 1.05-1.73), IC = 0.35 (95%CI: -0.02-0.70)] and etoposide [ROR = 4.65 (95%CI: 1.45-14.91), IC = 2.15 (95%CI: -0.25-2.74)] only met the positive ROR signal criteria, while IC signal did not.

## 4 Discussion

Based on FAERS database, this study systematically identified drug-related ILD safety signals in HNSCC treatment for the first time. Combined ROR and IC analysis confirmed that PD-1/PD-L1 inhibitors and anti-EGFR monoclonal antibodies are drugs with positive ILD safety signals, and the ILD association signal of PD-1/PD-L1 inhibitors is stronger than that of anti-EGFR monoclonal antibodies. This finding fills the gap in real-world studies on drug-related ILD in HNSCC.

PD-1/PD-L1 inhibitors and anti-EGFR monoclonal antibodies are core drugs for HNSCC. According to KEYNOTE-048, the former has replaced the latter as the first-line preferred option for HNSCC, while the latter still has irreplaceable clinical value in rapid tumor shrinkage, chemotherapy-free regimens, and late-line treatment (8). Since 2011, immunotherapy has emerged as a core pillar of cancer treatment, with PD-1/PD-L1 inhibitors being the most widely used immunotherapeutic drugs (9). Approximately 20-25% of patients achieve long−term benefits from immunotherapy (10–13). However, immune-related adverse events (irAEs) cannot be ignored. A meta-analysis including 125 trials showed that the overall incidence of irAEs reached 66.6%, and the incidence of grade ≥3 severe events was 14.0% (14). Although ILD is uncommon, it has a high fatal risk and is one of the most severe toxicities requiring vigilance. Its pathogenesis may involve immune tolerance breakdown, antigen cross-reactivity, and inflammatory factor-mediated lung injury. Thr reported incidence of ILD ranges from 2.6% to 33%, and prior thoracic radiotherapy and baseline lung diseases further increase the risk (15–19).

In terms of targeted therapy, more than 80% of HNSCC patients have EGFR overexpression. Cetuximab is the first anti-EGFR monoclonal antibody approved by the FDA for HNSCC treatment and plays an important role in the whole-course management (20). Its induced ILD may be related to immune-mediated alveolitis, direct drug-induced damage to alveolar epithelial cells, and inflammatory factor-mediated lung injury, but the specific mechanism has not been fully elucidated. Owing to data limitations, this study could not conduct subgroup analysis of PD-1/PD-L1 inhibitors plus cetuximab combinations, which needs further exploration. This study confirmed that, compared with targeted and immunotherapy, chemotherapeutic drugs had a weaker signal with ILD, and only etoposide showed a positive ROR but a negative IC, consistent with clinical cognition that chemotherapeutic drugs mainly act on rapidly dividing cells, and the lung tissue is not the main target. Of note, etoposide showed a positive ROR but a negative IC. This discrepancy stems from methodological differences: ROR is frequency-based and sensitive to small samples, whereas IC is a Bayesian measure requiring stable data for rare events. Insufficient samples or large data variation may lead to negative IC despite positive ROR. Extra caution should be exercised when interpreting such drug signals. Antibody-drug conjugates (ADCs) as novel antineoplastic drugs have been reported to have ILD risk in other tumors. A pooled analysis of trastuzumab deruxtecan (T-DXd) treatment showed that the incidence of ILD was 15.4%, of which 77.4% were grade ≤ 2, and onset mainly within 12 months (21). Currently, data on their pulmonary safety in HNSCC are limited and warrant further investigation.

In terms of onset time, drug-related ILD in HNSCC patients mostly occurs early, with a median onset of 32 days, and nearly 80% cases develop within three months. The median TTO of cetuximab and pembrolizumab is both 29 days, while that of nivolumab is slightly longer at 52 days. The shape parameter β of three drugs is < 1, suggesting that the early stage of medication is critical for ILD prevention. A previous study based on the Japanese Adverse Drug Event Report (JADER) database also reported a similar trend: 45 days for cetuximab, 56 days for nivolumab, and 40 days for pembrolizumab (22). In addition, previous studies have showed that the incidence of immunotherapy-related fatal adverse events is 0.3% to 1.3%, with an early onset (23, 24). This study also observed that the median onset time of fatal cases was slightly earlier than that of non-fatal cases, but the difference did not reach statistical significance.

Risk factors for drug-related ILD include advanced age, previous lung disease, history of thoracic radiotherapy, and combined medication (25). For example, age ≤ 60 years is associated with an increased frequency of PD-1/PD-L1 inhibitors-related pneumonitis (3). In the overall population analysis of this study, both nivolumab and pembrolizumab showed positive signals. However, within the subgroup of patients aged ≥65 years, only pembrolizumab retained a positive signal, whereas nivolumab, another agent of the same PD-1/PD-L1 inhibitor class, failed to present a significant positive signal. There are three potential reasons that can explain this difference. Firstly, these two drugs hold different clinical positions in HNSCC. With the support of the KEYNOTE-048 trial, pembrolizumab combined with chemotherapy can be used as the standard first-line treatment. Chemotherapy has pulmonary toxicity and synergically increases the risk of immune pneumonia. First-line patients have better physical condition and stronger immune response, therefore more prone to immune-mediated lung injury. Nivolumab is mostly used as a late-line monotherapy, and patients suffer from immune exhaustion. The risk of immune pneumonia decreases. At the same time, FAERS have reporting bias. The monitoring of the front-line population is more thorough, and the reporting of adverse reactions is more complete. In addition, the sample size of the elderly subgroup in this study is limited. This difference is only a preliminary signal and needs to be verified by a larger cohort. In healthcare professional reports, the signals of pembrolizumab and nivolumab were further strengthened, while cetuximab only met the ROR criterion. These findings demonstrate a more reliable association between immunotherapy and ILD. Further real-world and prospective studies are needed to confirm the correlation of cetuximab with ILD. Moreover, this highlights the value of high-quality reports and the necessity of combined ROR and IC analysis.

This study also has certain limitations. First, the FAERS database is a spontaneous reporting system, which inevitably suffers from issues such as reporting bias, underreporting, misreporting, and missing data. Second, the database only includes basic information such as age, gender, reporting country and indication, and lacks key clinical data closely related to HNSCC, including tumor stage, pre-existing lung disease, smoking history, and history of thoracic radiotherapy. Third, the high proportion of severe cases is largely attributable to the inherent reporting bias of the FAERS database, wherein severe and fatal events are preferentially reported while mild cases are underreported. Therefore, the proportions of severe outcomes and mortality observed in the present study should not be interpreted as true incidence rates or authentic risk estimates. Overall, data from spontaneous reporting systems can only be used for safety signal mining and cannot support causal inference. Further studies are needed to validate our findings.

In conclusion, this study detected the ILD safety signals of drugs for HNSCC treatment based on the FAERS database, which merit further validation. It is suggested that clinical practice should focus on pulmonary toxicity linked to anti-EGFR monoclonal antibodies and PD-1/PD-L1 inhibitors, and strengthen early monitoring and intervention.

## Data Availability

The datasets presented in this study can be found in online repositories. The names of the repository/repositories and accession number(s) can be found below: https://fis.fda.gov/extensions/fpd-qde-faers/fpd-qde-faers.html.
