# Supplementary material for: Disproportionality analysis of drug-related interstitial lung disease in patients with head and neck squamous cell carcinoma: a signal mining study
Source: Front Immunol. 2026 Jul 13;17:1858004. doi: 10.3389/fimmu.2026.1858004 (PMC13402548; doi:10.3389/fimmu.2026.1858004)
Supplement: Supplementary file 1 [file DataSheet1.pdf]

Supplementary Table 1. 2 × 2 Contingency Tables and Disproportionality Analysis Results for 42 Drugs Associated with ILD in HNSCC Patients

Drug	a	b	c	d	N	ROR(95% CI)	IC(95% CI)
Cetuximab	199	13863	341	34650	49053	1.46(1.22, 1.74)	0.36(0.12, 0.60)
Nivolumab	111	6508	429	42005	49053	1.67(1.35, 2.06)	0.61(0.30, 0.90)
Pembrolizumab	107	4552	433	43961	49053	2.39(1.93, 2.96)	1.06(0.74, 1.35)
Paclitaxel	23	1903	517	46610	49053	1.09(0.72, 1.66)	0.12(-0.50, 0.71)
Cisplatin	20	4657	520	43856	49053	0.36(0.23, 0.57)	-1.36(-1.97, -0.68)
Docetaxel	13	2386	527	46127	49053	0.48(0.27, 0.83)	-1.02(-1.76, -0.19)
Carboplatin	13	2369	527	46144	49053	0.48(0.28, 0.83)	-1.01(-1.75, -0.18)
Bevacizumab	6	701	534	47812	49053	0.77(0.34, 1.72)	-0.38(-1.43, 0.77)
Flurouracil	4	2074	536	46439	49053	0.17(0.06, 0.45)	-2.52(-3.56, -0.96)
Etoposide	3	60	537	48453	49053	4.51(1.41, 14.43)	2.11(-0.26, 2.72)
Lenvatinib	3	390	537	48123	49053	0.69(0.22, 2.15)	-0.53(-1.87, 1.04)
Durvalumab	3	184	537	48329	49053	1.47(0.47, 4.61)	0.54(-1.08, 1.84)
Bleomycin	2	6	538	48507	49053	30.05(6.05, 149.24)	4.51(-0.48, 3.38)
Afatinib	2	410	538	48103	49053	0.44(0.11, 1.75)	-1.18(-2.57, 0.79)
Tislelizumab	2	318	538	48195	49053	0.56(0.14, 2.27)	-0.82(-2.28, 1.08)
Ipilimumab	2	120	538	48393	49053	1.50(0.37, 6.08)	0.57(-1.34, 2.04)
Tiragolumab	2	12	538	48501	49053	15.03(3.35, 67.30)	3.7(-0.46, 3.19)
Cyclophosphamide	1	15	539	48498	49053	6.00(0.79, 45.49)	2.51(-1.41, 2.91)
Pemetrexed	1	248	539	48265	49053	0.36(0.05, 2.58)	-1.45(-2.96, 1.14)
Capecitabine	1	857	539	47656	49053	0.10(0.01, 0.73)	-3.24(-4.43, -0.34)
Vinorelbine	1	58	539	48455	49053	1.55(0.21, 11.21)	0.62(-1.81, 2.34)
Erlotinib	1	1001	539	47512	49053	0.09(0.01, 0.63)	-3.46(-4.64, -0.55)
Gefitinib	1	230	539	48283	49053	0.39(0.05, 2.78)	-1.35(-2.88, 1.22)
Crizotinib	1	55	539	48458	49053	1.63(0.23, 11.83)	0.7(-1.78, 2.38)
Ribociclib	1	4	539	48509	49053	22.50(2.51, 201.64)	4.18(-1.45, 3.27)
Palbociclib	1	314	539	48199	49053	0.28(0.04, 2.03)	-1.79(-3.22, 0.89)
Lapatinib	1	145	539	48368	49053	0.62(0.09, 4.43)	-0.68(-2.45, 1.67)

Copanlisib	1	22	539	48491	49053	4.09(0.55, 30.39)	1.98(-1.47, 2.79)
Cabozantinib	1	287	539	48226	49053	0.31(0.04, 2.22)	-1.66(-3.12, 0.99)
Pazopanib	1	147	539	48366	49053	0.61(0.09, 4.37)	-0.7(-2.46, 1.66)
Isatuximab	1	0	539	48513	49053	-	6.51(-1.92, 3.86)
Trastuzumab	1	32	539	48481	49053	2.81(0.38, 20.61)	1.46(-1.56, 2.64)
Panitumumab	1	159	539	48354	49053	0.56(0.08, 4.04)	-0.82(-2.53, 1.58)
Toripalimab	1	274	539	48239	49053	0.33(0.05, 2.33)	-1.6(-3.07, 1.04)
Tremelimumab	1	6	539	48507	49053	15.00(1.80, 124.80)	3.7(-1.41, 3.16)
Bortezomib	1	265	539	48248	49053	0.34(0.05, 2.41)	-1.55(-3.03, 1.07)
Vismodegib	1	68	539	48445	49053	1.32(0.18, 9.54)	0.4(-1.90, 2.25)
Niraparib	1	46	539	48467	49053	1.95(0.27, 14.20)	0.95(-1.70, 2.47)
Figitumumab	1	2	539	48511	49053	45.00(4.07, 497.05)	4.92(-1.56, 3.44)
Methylprednisolone	1	22	539	48491	49053	4.09(0.55, 30.39)	1.98(-1.47, 2.79)
Amifostine	1	160	539	48353	49053	0.56(0.08, 4.01)	-0.83(-2.53, 1.58)
Folinic acid	1	59	539	48454	49053	1.52(0.21, 11.02)	0.6(-1.82, 2.33)

a: number of reports with the drug and ILD in HNSCC patients; b: number of reports with the drug but without ILD in HNSCC patients; c: number of reports with ILD but without the drug in HNSCC patients; d: number of reports without the drug and without ILD in HNSCC patients; N: total reports of the 42 enrolled drugs in HNSCC patients; ROR: reporting odds ratio; IC: information component; CI: confidence interval; HNSCC: head and neck squamous cell carcinoma; ILD: interstitial lung disease.
